# Novel unbiased equations to calculate triglyceride-rich lipoprotein cholesterol from routine non-fasting lipids

**DOI:** 10.1186/1475-2840-13-56

**Published:** 2014-03-10

**Authors:** Michel P Hermans, Sylvie A Ahn, Michel F Rousseau

**Affiliations:** 1 Division of Endocrinology & Nutrition, Cliniques universitaires St-Luc and Institut de Recherche Expérimentale et Clinique (IREC), Université catholique de Louvain, Brussels, Belgium; 2 Division of Cardiology, Cliniques universitaires St-Luc and Pôle de Recherche Cardiovasculaire, Institut de Recherche Expérimentale et Clinique (IREC), Université catholique de Louvain, Brussels, Belgium; 3 DipNatSci DipEarthSci DipGeogEnv PGCert (SocSc), Endocrinology & Nutrition, UCL 54.74 Tour Claude Bernard +1, avenue Hippocrate 54, Brussels B-1200, Belgium

**Keywords:** Remnant cholesterol, Triglycerides, Non-fasting, Chylomicrons, Lipoprotein, Diabetes, Atherogenic dyslipidemia

## Abstract

**Background:**

Non-fasting triglyceride-rich lipoproteins cholesterol (TRL-C) contributes to cardiovascular risk, in that it includes remnant cholesterol (RC). TRL-C is computed as total C - [LDL-C + HDL-C]. Such calculation applies only if LDL-C is directly measured, or obtained from a non-Friedewald’s formula, a method as yet never benchmarked against independent markers of TRL burden.

**Methods:**

The *Discriminant Ratio* (DR) methodology was used in 120 type 2 diabetic patients in order: (*i*) to compute TRL-C from non-fasting lipids; (*ii*) to establish the performance of TRL-C and TRL-C/apoA-I (*vs.* TG-based markers) to grade TRLs and atherogenic dyslipidemia (AD); and (*iii*) to relate TRL-C with non-fasting TG.

**Results:**

Depending on apoB_100_ availability, TRL-C (*mg/dL*) can be derived from non-fasting lipids in two ways: (*a*) total cholesterol (TC) - [(0.0106 * TC - 0.0036 * TG + 0.017 * apoB_100_ - 0.27) * 38.6] - HDL-C; and (*b*) TC - [(0.0106 * TC - 0.0036 * TG + 0.017 * [0.65 * (TC - HDL-C) + 6.3] - 0.27) * 38.6] - HDL-C. Discrimination between *log*[TG] and TRL-C was similar (DR 0.94 and 0.84, respectively), whereas that of *log*[TG]/HDL-C was better than TRL-C/apoA-I (DR 1.01 *vs.* 0.65; *p* 0.0482). All Pearson’s correlations between pairs reached unity, allowing formulation of two unbiased equivalence equations: (*a*) TRL-C = 97.8 * *log*[TG] - 181.9; and (*b*) TRL-C/apoA-I = 8.15 * (*log*[TG]/HDL-C) - 0.18.

**Conclusions:**

TRL-C and *log*[TG] are as effective and interchangeable for assessing remnant atherogenic particles. For grading TRL-AD, it is best to use *log*[TG]/HDL-C, inherently superior to TRL-C/apoA-I, while measuring the same underlying variable.

## Introduction

Cholesterol (C) in atherogenic particles other than low-density lipoproteins (LDL) is an emerging risk factor (RF) for ischemic heart disease, and is mostly found in fasting and non-fasting triglyceride-rich lipoproteins (TRLs). TRLs comprise two clusters: (*i*) chylomicrons (CM; triglycerides (TG)-rich lipoproteins (TRLs) of intestinal origin) and their remnants (CMR), each carrying one apolipoprotein (apo) B_48_); and (*ii*) very-low density lipoproteins (VLDL; endogenous TRLs which originate in the liver, and their relatively TG-depleted remnants (VLDL-R), each carrying one apoB_100_) [1-13].

Table 1 describes the distribution of fasting and nonfasting lipid particles, TRLs, and TRL remnants distribution, with respective contribution to total cholesterol, TRL-cholesterol (TRL-C), and TRL-remnant cholesterol (TRL-RC), alongside their major corresponding apolipoprotein(s). Among TRLs, nonfasting residual TG-rich particles are considered as a major contributor to residual vascular risk (RVR), even in patients on statins whose LDL-C reaches target. These residual lipoproteins are a blend of chylomicrons remnants (CM-R) and very-low-density lipoprotein remnants (VLDL-R). Their atherogenicity is related to their ability to deliver cholesterol in vessels walls.

**Table 1:** Fasting and nonfasting lipid particles, triglyceride-rich lipoproteins (TRL), and TRL remnants distribution, with respective contribution to total cholesterol, TRL-cholesterol and TRL-remnant cholesterol, alongside major corresponding apolipoprotein(s)

**Fasting**			**Nonfasting**		
**All lipoproteins**	**Total cholesterol**	**Apolipoprotein(s)**	**All lipoproteins**	**Total cholesterol**	**Apolipoprotein(s)**
(CM-R)	(CM-R-C)	(apoB_48_)	CM	CM-C	apoB_48_
VLDL	VLDL-C	apoB_100_	CM-R	CM-R-C	apoB_48_
VLDL-R	VLDL-R-C	apoB_100_	VLDL	VLDL-C	apoB_100_
IDL	IDL-C	apoB_100_	VLDL-R	VLDL-R-C	apoB_100_
LDL	LDL-C	apoB_100_	IDL	IDL-C	apoB_100_
HDL	HDL-C	apoA-I; apoA-II	LDL	LDL-C	apoB_100_
Lipoprotein(a)	Lipoprotein(a)-C	apoB_100_; apo(a)	HDL	HDL-C	apoA-I; apoA-II
			Lipoprotein(a)	Lipoprotein(a)-C	apoB_100_; apo(a)
**TRLs**	**TRL-cholesterol**	**Apolipoprotein(s)**	**TRLs**	**TRL-cholesterol**	**Apolipoprotein(s)**
(CM-R)	(CM-R-C)	(apoB_48_)	CM	CM-C	apoB_48_
VLDL	VLDL-C	apoB_100_	CM-R	CM-R-C	apoB_48_
VLDL-R	VLDL-R-C	apoB_100_	VLDL	VLDL-C	apoB_100_
			VLDL-R	VLDL-R-C	apoB_100_
**TRL remnants**	**TRL-remnant cholesterol**	**Apolipoproteins(s)**	**TRL remnants**	**TRL-remnant cholesterol**	**Apolipoprotein(s)**
(CM-R)	(CM-R-C)	(apoB_48_)	CM-R	CM-R-C	apoB_48_
VLDL-R	VLDL-R-C	apoB_100_	VLDL-R	VLDL-R-C	apoB_100_

*apo* apolipoprotein, *C* cholesterol, *CM* chylomicron *CM-R* chylomicron remnant, *HDL* high-density lipoprotein, *IDL* intermediate-density lipoprotein, *LDL* low-density lipoprotein, *TRL* triglyceride-rich lipoprotein, *VLDL* very-low density lipoprotein, *VLDL-R* very-low density lipoprotein remnant; parentheses refer to very low presence.

Quantification of the cholesterol content of TG-rich particles would be informative to better characterize their nonfasting atherogenic load. However this would require direct specific lipoproteins measurement beyond routine clinical practice. For this reason, several authors recently proposed to use a simple formula to measure “remnant cholesterol” (RC) from standard nonfasting lipids, “RC” being calculated as total C - (high-density-lipoprotein (HDL)-C + LDL-C) [10,11]. Such equation however measures TRL-C (the sum of CM-C, CMR-C, VLDL-C and VLDL-R-C), and not RC. The latter is the sum of CMR-C and VLDL-R-C, and accounts for only two out of four components of TRL-C (Table 1).

As for RC, non-fasting TRL-C is usually not measured, but derived according to the formula set out above: TRL-C = total C *minus* [LDL-C + HDL-C] [8-11]. Whereas LDL-C can be inferred from Friedewald’s equation in fasting conditions, this calculation underestimates LDL-C in moderate to severe hypertriglyceridemia (200-400 mg/dL), and is inapplicable for fasting TG >400 mg/dL [14]. To overcome this inaccuracy, a novel method for estimating LDL-C from standard lipid profile using an adjustable factor for the TG:VLDL-C ratio was recently proposed by Martin *et al*. [15,16].

By its very nature, Friedewald’s equation is unusable in the non-fasting state. This is because in the presence of elevated fasting TG or in non-fasting conditions, the C-to-TG content of remnant lipoproteins (RLPs) infringes the steady 1/5 ratio (20% C and 80% TG) at the core of Friedewald’s method [14-20]. Determining non-fasting TRL-C using a Friedewald-derived LDL-C level is methodologically inadequate, as it merely equates TRL-C with one-fifth of non-fasting TG, thus failing to provide additional information beyond TG levels [8,10,11].

The lack of a simple and suitable method to determine TRL-C currently limits its clinical use for evaluating RVR, unless adequate alternatives to determine nonfasting LDL-C are used. To circumvent this difficulty, an alternative approach is to derive LDL-C from apoB_100_, waiving the bias generated by applying a fixed C-to-TG ratio that assumes constant and identical composition for all RLPs. A disadvantage of this method is the requirement for direct apoB_100_ measurement, limiting its use unless apoB_100_ is inferred from routine lipids, as previously described [17-21].

The aims of this study were: (*i*) to provide relevant equations to estimate TRL-C from non-fasting lipids, regardless apoB_100_ availability; (*ii*) to establish the performance and equivalence of TG-based markers *vs.* TRL-C and TRL-C/apoA-I, a continuous estimator of atherogenic dyslipidemia (AD); and (*iii*) to derive an unbiased equation predicting TRL-C from non-fasting TG. We used the *Discriminant Ratio* (DR) methodology which standardizes comparisons between measurements by taking into account fundamental properties for assessing imprecision and practical performance of tests designed to quantify similar variables [19,22,23].

## Methods

We studied 120 consecutive (86% white Caucasians) patients with T2DM, treated or not with lipid-lowering drug(s) (LLD). Age; gender; diabetes duration; smoking history; anthropometric indices; hypertension and metabolic syndrome (MetS) prevalence, the latter defined according to the harmonized criteria of Alberti *et al.*[24]; habitual ethanol intake; current glucose-lowering drugs, and LLDs were analysed. Hypertension was defined as systolic ≥140 and/or diastolic blood pressure ≥90 mmHg, and/or treatment with antihypertensive drugs. Glomerular filtration rate was estimated (eGFR) using the *Modification of Diet in Renal Disease* formula [25]. Albuminuria was defined as an albumin excretion ≥30 μg.mg creatinine^-1^.1.73 m^2^ from first-morning sample. Coronary and peripheral artery disease (CAD and PAD) were diagnosed as in [26], while stroke was defined according to *UK Prospective Diabetes Study* (UKPDS) criteria [27]. Atherogenic dyslipidemia (AD) was defined according to [28-30].

The following variables were measured in the non-fasting state: glycated haemoglobin (HbA_1c_), total C; HDL-C; TG; apoA-I and apoB_100_, with total C and TG determined with SYNCHRON system (Beckman Coulter Inc., Brea, CA); HDL-C with ULTRA-N-geneous reagent (Genzyme Corporation, Cambridge, MA); apoA-I and apoB_100_ by immunonephelometry (BNII Analyzer, Siemens Healthcare Products GmbH, Marburg, Germany). Nonfasting routine lipids, apoA-I and apoB_100_ were measured on two non-consecutive days for DR calculation, with a 4-6 months’ interval between samples. A non-HDL-C/apoB_100_ ratio >2.6 was an exclusion criterion because highly suggestive for Type III hyperlipoproteinemia [31]. The within-subjects coefficients of variation were: 5.4% [total C]; 7.1% [HDL-C]; 4.9% (apoA-I) and 6.9% [apoB_100_].

TRL-C was calculated from non-fasting lipids by subtracting [LDL-C + HDL-C] from total C, with LDL-C computed using Planella’s formula [17,19]:

$$\begin{array}{ll} \mathrm{LDL}‒C\left(\mathrm{mmol}/L\right)= & 0.41*\mathrm{TC}\left(\mathrm{mmol}/L\right)-0.32\\ & *\mathrm{TG}\left(\mathrm{mmol}/L\right)+1.7\\ & *\mathrm{apo}B_{100}\left(g/L\right)-0.27 \end{array}$$

Normal TRL-C values from 50 apparently-healthy lean Caucasians, untreated with LLD and without familial hypercholesterolemia or early-onset parental CV disease, were (*mg/dL*): 24 (*mean*); 21 (*median*); 17 (*SD*); 2 (*minimum*); 71 (*maximum*); 13 (*percentile 25*) and 29 (*percentile 75*).

Each patient gave written informed consent; the study was performed in agreement with Helsinki’s Declaration; Good Clinical Practice principles; and the local Institutional Review Board.

## Statistics

The *Discriminant Ratio* (DR) methodology compares different tests measuring the same underlying physiological variable by determining the ability of a test to discriminate between different subjects, and the comparison of discrimination between different tests as well as the underlying correlation between pairs of tests adjusting for attenuation due to within-subject variation [22]. In a comparison study where duplicates measurements are performed in each subject, the measured between-subject standard deviation (SD_B_) is calculated as the SD of the subject mean values calculated from the 2 replicates.

•The standard mathematical adjustment to yield the *underlying* between-subject SD (SD_U_) is: SD_U_ = √ (SD^2^_B_ - SD^2^_W_/2);

•The *within*-subject variance (V_w_) calculated (for *m* repeat tests) as (V_w_) = Σ(x_j_ -x_i_ )^2^/(m-1)), the within-subject SD (SD_w_) being its square root;

•The DR represents the ratio SD_U_/SD_W_

Confidence limits for DR’s and the testing for equivalence of different DR’s were calculated and differences were considered significant for *p* < 0.05. Given sample size and number of replicates, the minimal detectable significant difference in DR for the present study was 0.42. Coefficients of correlation between pairs of tests (measured *vs.* estimated) were adjusted to include an estimate of the underlying correlation, as standard coefficients tend to underestimate the true correlation between tests, due to within-subject variation [22].

Results are presented as means (±1 standard deviation [SD]), or as proportions (%). The significance of differences between means was assessed by Student’s *t* test, or by Welch’s test for data sets with significant differences in SDs, and by Chi^2^ test for differences in proportions. Results were considered statistically significant or non-significant (NS) for *p* <0.05 or *p* ≥0.05, respectively.

## Results

Patients’ characteristics are described in Table 2. Mean age (1 SD) was 67 (11) years, with a male gender predominance. Mean body mass index was in the overweight range. Patients had long-standing diabetes (mean duration 16 (9) years), with a majority also having hypertension (89%), and a MetS phenotype (92%). Current smokers amounted to 13%; habitual ethanol intake was 10 (18) U/week. Mean glycaemic control, as reflected by HbA_1c_, was suboptimal at 7.79 (1.32)% (62 (10) mmol/mol). Overall microangiopathy prevalence was 58%: retinopathy 37%; polyneuropathy 28% and/or (micro) albuminuria 29%.

**Table 2:** Patients’ characteristics

**n**		**120**
Age	Years	67 (11)
Diabetes duration	Years	16 (9)
Male: female	%	63: 37
Smoking^§^		42-45-13
Body mass index	kg.m^-2^	30.2 (5.6)
Waist circumference	cm	106 (15)
Metabolic syndrome	%	92
Hypertension	%	89
Anti-dyslipidemic drug(s)	%	92
Statin-fibrate-ezetimibe	%	81-38-12
HbA_1c_	%	7.79 (1.32)
HbA_1c_	mmol.mol^-1^	62 (10)
Glomerular filtration rate	mL.min^-1^ 1.73 m^2^	73 (27)
Albuminuria	μg.mg creatinine^-1^	90 (240)
Atherogenic dyslipidemia	%	58
Total cholesterol	mg.dL^-1^	172 (34)
Non-HDL-C	mg.dL^-1^	127 (33)
LDL-C	mg.dL^-1^	91 (26)
TRL-C	mg.dL^-1^	36 (20)
TG	mg.dL^-1^	197 (101)
*log*[TG]	mg.dL^-1^	2.23 (0.20)
apoB_100_	mg.dL^-1^	89 (21)
apoA-I	mg.dL^-1^	145 (25)
HDL-C	mg.dL^-1^	44 (12)
*log*[TG]/HDL-C		0.057 (0.024)
TRL-C/apoA-I		0.28 (0.20)
Macroangiopathy	%	33
Coronary artery disease	%	23
Peripheral artery disease	%	11
Transient ischemic attack/stroke	%	7

Results are expressed as means (1 SD) or proportions, with nonfasting lipids and lipoproteins values representing the means of Day 1 and Day 2. *Apo* apolipoprotein, *C* cholesterol, *HbA*_*1c*_ glycated haemoglobin, *HDL* high-density lipoprotein, *LDL* low-density lipoprotein, *TG* trigylycerides, *TRL* TG-rich lipoprotein; ^§^never-former-current.

Most patients were on LLDs: statins (81%) and/or fibrates (38%). Current mean lipids and lipoproteins values were illustrative of patients with the usual form of T2DM, i.e. associated with central adiposity, insulin resistance (IR) and MetS: low HDL-C with raised non-HDL-C, apoB_100_, TG and TRL-C, together with a high prevalence of AD (58%). Overall macroangiopathy prevalence was 33%: CAD [23%]; PAD [11%] and/or cerebrovascular disease [7%].

**Figure 1:**
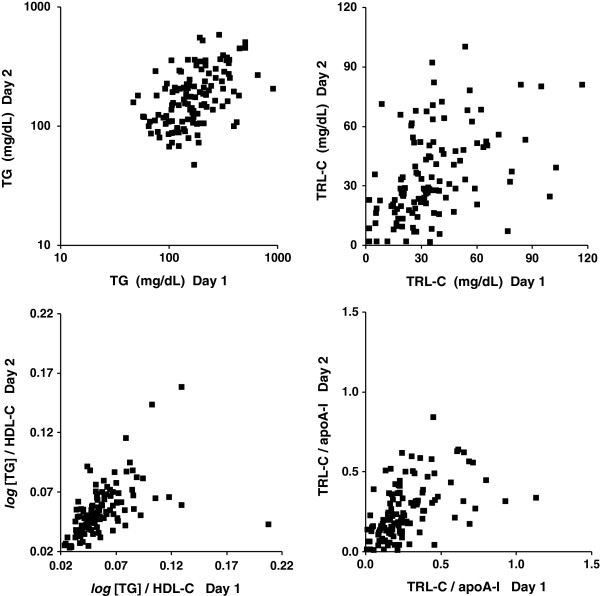
**Plots of untransformed values obtained on day 1 (*X axis*) and day 2 (*Y axis*) for nonfasting triglycerides (TG) (*upper left*); TG-rich lipoprotein cholesterol (TRL-C) (*upper right*); nonfasting *log *** **[TG]/high-density lipoprotein cholesterol (HDL-C) (*lower left*); and TRL-C/apolipoprotein (apo) A-I (*lower right*) in 120 patients with type 2 diabetes.**

Figure 1 shows the plots of untransformed values on two different days for TG; TRL-C; *log*[TG]/HDL-C; and TRL-C/apoA-I, respectively. Relative median day-to-day variations were: 36% (TG); 39% (TRL-C); 16% (*log*[TG]/HDL-C); and 46% (TRL-C/apoA-I), respectively. Figure 1 also shows the heteroscedastic arrangement of the data spread on repeat measurements.

The precision, discrimination and interrelation of each non-fasting estimate, expressed as underlying between-subject standard deviation (SD_u_), global within-subject standard deviation (SD_w_), discriminant ratio (DR); and measured Pearson’s product-moment correlation coefficients are shown in Table 3. For the *log*[TG] *vs.* TRL-C comparison, the respective SD_u_/SD_w_ (DRs) were 0.94 and 0.84, and the difference in discriminatory power between the two determinations did not reach statistical significance. As regards the *log*[TG]/HDL-C *vs.* TRL-C/apoA-I comparison, the respective DRs were 1.01 and 0.65, the discriminatory power of *log*[TG]/HDL-C being significantly better than of TRL-C/apoA-I (*p* 0.0482).

**Table 3:** **Precision and discrimination of nonfasting TG, TRL-C, and atherogenic dyslipidemia ratios expressed as *between-subject Standard Deviations *** **(SD**_**u**_**), *global within subject Standard Deviation *** **(SD**_**w**_**), *Discriminant Ratio *** **(DR), and measured Pearson’s correlation coefficients between pairs of variables [adjusted for attenuation]**

	**SD**_**u**_	**SD**_**w**_	**DR**	**CIs**	***p***	**Pearson’s coefficient**	
*log*[TG]	**0.161**	**0.171**	**0.94**	[0.72-1.19]	*0.5604*	**0.92**	[1.00]
TRL-C	**15.20**	**18.06**	**0.84**	[0.61-1.09]			
*log*[TG]/HDL-C	**0.0198**	**0.0195**	**1.01**	[0.79-1.27]	*0.0482*	**0.89**	[1.00]
TRL-C/apoA-I	**0.138**	**0.210**	**0.65**	[0.40-0.90]			

Values from nonfasting results of individual tests and means of their duplicates in 120 type 2 diabetes patients, with [2.5 - 97.5%] confidence intervals (CIs) for DR’s. *Apo* apolipoprotein, *C* cholesterol, *HDL* high-density lipoprotein, *TG* triglycerides, *TRL* TG-rich lipoprotein. P values represented the significancies of the differences between DRs of each pair of variables. For correlation between estimates, values were obtained from the mean of different measurements performed on separate days.

The Pearson’s correlations between each pair of tests were high, respectively 0.92 (*log*[TG] *vs.* TRL-C) and 0.89 (*log*[TG]/HDL-C *vs.* TRL-C/apoA-I), each correlation reaching unity, once values were adjusted for attenuation prior to correlation (Table 3).

**Figure 2:**
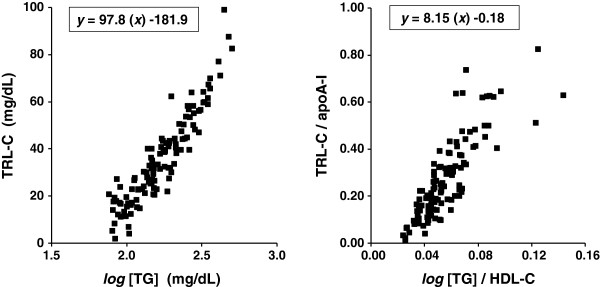
**Equivalence between tests.***Left panel*: plots of untransformed values of *log*[triglycerides (TG)] (*X axis*) *vs*. TG-rich lipoprotein cholesterol (TRL-C) (*Y axis*). *Right panel*: plots of *log*[TG]/high-density lipoprotein cholesterol (HDL-C) (*X axis*) *vs*. TRL-C/apolipoprotein (apo) A-I (*Y axis*). The equations of the two unbiased lines of equivalence relating each pair of measurements are provided on the graphs. All values obtained from the means of nonfasting values measured on two different days in 120 patients with type 2 diabetes mellitus.

Figure 2 shows the plots of untransformed values (means of Day 1 and Day 2) for *log*[TG] *vs*. TRL-C, and *log*[TG]/HDL-C *vs.* TRL-C/apoA-I. The equations of the unbiased lines of equivalence relating each pair of tests were:

$$\mathrm{TRL}‒C\left(\mathrm{mg}/\mathrm{dL}\right)=98*\log\left[\mathrm{non}‒\mathrm{fasting}\;\mathrm{TG}\right]\;\left(\mathrm{mg}/\mathrm{dL}\right)-182$$

$$\mathrm{TRL}‒C/\mathrm{apoA}‒I=8.15*\left(\log\left[\mathrm{non}‒\mathrm{fasting}\;\mathrm{TG}\right]/\mathrm{HDL}‒C\right)-0.18$$

Practically, these equations allow for calculating TRL-C without bias from standard non-fasting lipids, depending on apoB_100_ availability (units mg/dL):

1. *apoB*_*100*_*is available alongside standard non-fasting lipids*:

$$\begin{array}{ll} \mathrm{TRL}‒C=\mathrm{TC} & -\left[\left(0.0106\;*\;\mathrm{TC}-0.0036\;*\;\mathrm{TG}+0.017\right)\right)\\ & \left(\left(*\;\mathrm{apo}B_{100}-0.27\right)\;*\;38.6\right]-\mathrm{HDL}‒C \end{array}$$

2. *apoB*_*100*_*level is not available, and is computed according to*[19]:

$$\begin{array}{ll} \mathrm{TRL}‒C=\mathrm{TC} & -\left[\left(0.0106\;*\;\mathrm{TC}-0.0036\;*\;\mathrm{TG}+0.017\right)\right)\\ & \left(*\;\left[0.65\;*\;\left(\mathrm{TC}‒\mathrm{HDL}‒C\right)+6.3\right]-0.27\right)\\ & \left(*\;38.6\right]-\mathrm{HDL}‒C \end{array}$$

## Discussion

Measuring TG represents an easy means to estimate the combined mass of fasting or non-fasting TRLs, as surrogate for their cholesterol load. An excess of TRLs, including RLPs, throughout the nycthemeron epitomizes CMR conditions such the MetS, IR and the common form of T2DM. Elevated levels of TRL-C contribute, alongside LDL-C, to plaque formation and progression. TRL-C is a modifiable driver of RVR. Whereas TG as such are not atherogenic, the well-demonstrated association between fasting or non-fasting TG and CVD is underlied by the atherogenicity of TRLs, especially that of RLPs [2-6,8-11,32,33].

In *fasting conditions*, hypertriglyceridemia >150 mg/dL is categorized as “elevated” VLDL, corresponding to TRL-C levels >30 mg/dL. Such assumption of equivalence is valid only if the composition of VLDL, in terms of C and TG, is in a ratio of 1/5. This is not necessarily the case in CMR states where non-VLDL TRLs (among which numerous RLPs) co-exist alongside VLDL. Likewise, in *non-fasting conditions*, TRLs are further heterogeneous, in size and composition, being populated by various TG-enriched and relatively TG-depleted lipoproteins (including remnants from the endogenous and exogenous pathways). The C/TG ratio of non-fasting TRLs substantially differs from that of fasting TRLs, the latter essentially consisting of standard VLDL with 20% cholesterol [1,3,13-17,20,21].

For this reason, recent articles on the usefulness of “RC” as residual risk marker relied by default on Friedewald’s formula to estimate the LDL-C component of the equation. Doing so, their authors did not distinguish TRL-C from RC. Such an oversimplification ascribes to RC all the observed risk of non-fasting TRL-C. Besides, their rationale for extending the use of Friedewald’s equation to determine LDL-C in non-fasting samples, in place of a direct assay, relied upon a linear relationship between calculated and measured LDL-C in a reference subgroup, such a relationship being a self-fulfilling prophecy [8,10,11,14].

The present results provide unbiased and physiologically-consistent equations to determine TRL-C from non-fasting lipids, regardless apoB_100_ availability. As expected, non-fasting TRL-C and *log*[TG] were highly correlated, with adjusted Pearson’s coefficient reaching unity. Since both measures have uniform precision and discrimination, they provide similar information for ranking patients according to non-fasting TRLs, and are interchangeable. Yet, conceptually and educationally, determining TRL-C as surrogate for TRLs is more attractive, since it quantifies the atherogenic component directly involved in driving CV risk, including all the cholesterol load from RLPs. In this context, the DR method provides an unbiased equivalence equation allowing to predict TRL-C from *log*[TG], or *vice-versa*.

While the concept of low HDL-C as unconditional RF is strongly debated, the coexistence of elevated fasting TG together with low fasting HDL-C allows identifying patients with AD, in whom residual risk is particularly high, even when on-statin LDL-C is controlled [28-30,33,34]. An explanation for the accrued RVR from AD in the fasting state is that it could be a marker for high numbers of postprandial TRLs and elevated non-fasting TRL-C. This is supported by results from an ACCORD-Lipid sub-study, in which fenofibrate similarly lowered non-fasting TG in all T2DM participants, while reducing postprandial apoB_48_ excursions only in individuals with elevated fasting TG at baseline, a subgroup in which fenofibrate reduced CV outcomes [35].

In the presence of fasting hypertriglyceridemia (>150 mg/dL), the HDL-C cutoffs defining AD (≤40 mg/dL [men] and ≤50 mg/dL [women]) are transposable to non-fasting conditions, because remnant TRLs have little influence on HDL-C. Contrariwise, there are currently no standards or agreement defining (*i*) the upper physiological value for non-fasting TG; (*ii*) the sampling time after meal; and (*iii*) the lipid content and composition of the previous meal. For all these reasons we suggest to use either [TRL-C/apoA-I] or [*log*[TG]/HDL-C] to assess postprandial AD as a continuous variable.

As the underlying correlation between TRL-C and *log*[TG] on one hand, and between TRL-C/apoA-I and *log*[TG]/HDL-C on the other hand, reached unity once pre-analytical and analytical attenuation were taken into account, these two approaches may be used interchangeably to assess equivalent biological measures. While there was no significant difference between the discriminating performance of *log*[TG] compared to TRL-C, the discrimination of the ratio *log*[(TG]/HDL-C was clearly and significantly higher than the TRL-C/apoA-I ratio to quantify the severity of non-fasting AD in patients at high CMR. Given the perfect concordance between pairs of measurements, the clinician may prefer to expressing CV risk linked to TRL-C (intuitively more educational than *log*[TG]), and to determine CV risk related to AD by calculating *log*[TG]/HDL-C, which is superior to TRL-C/apoA-I. The latter has the inconvenience to require apoA-I determination on top of routine lipids. Regarding biometric equivalence between atherogenic-antiatherogenic ratios, we previously reported that non-HDL-C/HDL-C provides CV risk stratification similar to the apoB_100_/apoA-I ratio [36].

In this study, the performance of the above measures to that of a direct measurement of RC was not assessed, since the latter is not part of routine risk assessment. As regards cohort’s size, we compared the performance of two means to assess the burden of atherogenic TRL in 120 patients, an ample number given the DR methodology, which only requires ≥20 samples with 2 replicates as long as they represent a clinically-meaningful range for the variable under study [*see Appendix of*[22]*for a detailed discussion on sample size requirements for estimating DRs*]. The fact that patients had T2DM in this study does not limit the applicability of the findings, since the metabolic and pathophysiological fundamentals of TRL, CMR and RC are similar in diabetic and nondiabetic subjects, at increasing levels along a *continuum*, from normal to impaired fasting glucose, and from prediabetes to T2DM [37,38].

In conclusion, estimating TRL-C requires formulas which reflect the complex compositional changes in non-fasting TRLs, the latter consisting of particles not exclusively generated along the VLDL pathway, in which TG-content is heterogeneous and changes dynamically. We provide two unbiased equations to estimate the burden of TRL-C based on routine nonfasting lipids, depending on apoB_100_ measurement availability. Our results show that TRL-C and *log*[TG] are as effective and interchangeable to assess the atherogenic load of nonfasting TRLs. However, to grade TRL-related AD, it is better to use *log*[TG]/HDL-C, which is inherently superior to TRL-C/apoA-I, while measuring the same underlying variable.

## Competing interest

The authors declare that they have no competing interest.

## Authors’ contribution

All authors have read and approved the manuscript.
